# Effects of surface curvature on electron Monte Carlo (eMC) calculation results

**DOI:** 10.1002/acm2.14020

**Published:** 2023-05-04

**Authors:** Jonathan A. Polignani

**Affiliations:** ^1^ St. Mary's Hospital New York New York USA

**Keywords:** curved patient surfaces, electron beam physical effects, electron curvature, electron monitor unit verification, electron monte carlo, electron radiotherapy, electron surface contour, Varian eMC

## Abstract

Some clinical situations in radiotherapy require electron beams to be incident on curved patient surfaces. This study presents central‐axis dose output (cGy/MU) and percent dose versus depth (PDD) data that show the effects of curvature on results computed by the Varian eMC v15.6 algorithm using 6, 9, 12, 16, and 20 MeV electron beams incident on virtual phantoms with curved surfaces. The phantoms were designed to simulate common treatment sites. The dose outputs at the depth of maximum dose (dmax) on the central axis were observed to decrease 0%–14%, and several features of the PDDs for the A10 applicator changed, including up to 12% increased entrance dose. These dosimetric changes have the greatest effect on treatment sites with a radius of curvature of 10 cm or less, such as the scalp, nose, neck, and extremities. The concept of applying a curvature correction factor based on relative output data is presented to help clinical users mitigate discrepancies between calculations performed by simple monitor unit verification systems and accurate treatment planning dose algorithms.

## 1 INTRODUCTION

**FIGURE 1:**
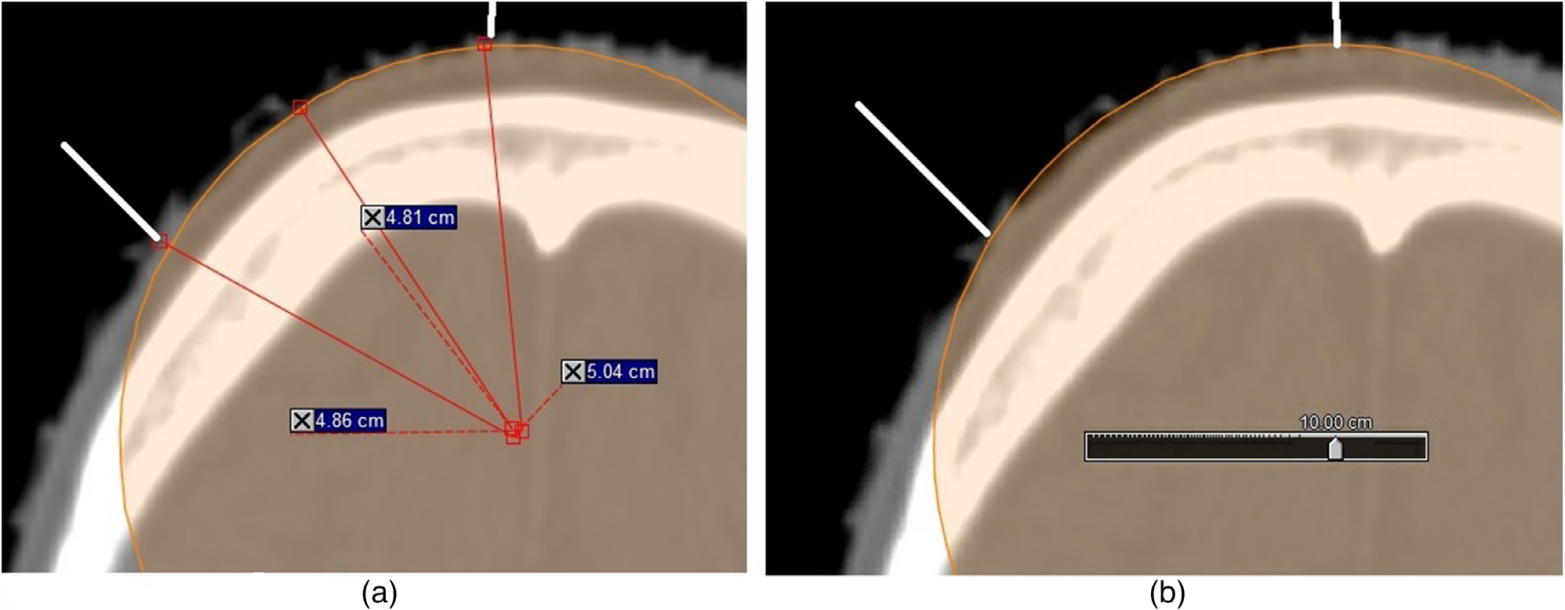
(a and b): Axial slices of a clinical example of a right anterior cranial case. The white lines indicate the field boundaries on the skin. The radius of curvature can be determined by drawing lines perpendicular to the surface to find an intersection point equidistant from several points on the surface (a), or by using a circular brush contouring tool displaying the diameter; the slider reads “10.00 cm” (b). The radius of this example can be approximated as 5 cm.

Many resources recommend that electron beam dosimetry be performed in water or solid‐water phantoms, which have a flat surface.^1^, ^2^, ^3^, ^4^, ^5^, ^6^ However, many clinical fields used in electron radiotherapy are directed onto surfaces that exhibit curvature in two or three dimensions. Treatment sites that exhibit curvature include the extremities, cranium, neck, breast, chest, chest wall, thorax, abdomen, and others (Table 1).^2^, ^7^, ^8^, ^9^, ^10^, ^11^, ^12^, ^13^, ^14^, ^15^, ^16^ Curved surfaces of the patient may change or appear irregular, and some curvature may be reduced by applying bolus. Still, many anatomical sites can be approximated by a regular curved surface such as the surface of a cylinder or sphere (Figure 1).

**TABLE 1:** Examples of clinical radii of curvature for surfaces of different anatomical sites.

**Site**	**Possible radii of curvature (cm)**	**References**
Chest wall	10–20	Ref. 7
Scalp, cranium	5–10	See Figure 1; Ref. 8
Breast	5–15	Ref. 7
Nose	1–3	Refs. 2, 9, 10
Calf, forearm	3–7	Refs. 2, 11, 12
Neck	2.5–7	Refs. 13, 14, 15, 16

Curvature is a type of surface irregularity. However, previous discussions of surface irregularities have addressed: (1) angled beam incidence upon a flat surface, (2) developing conformal bolus or compensation techniques to reduce surface irregularities and improve dose homogeneity for sites with irregular surfaces such as the nose or ear, and (3) irregularities in the proximal surface of an electron conformal therapy bolus that result in hot and cold spots.^10^, ^17^, ^18^, ^19^, ^20^, ^21^, ^22^ There is limited prior investigation focused on the dosimetric effects of clinical electron beams incident perpendicularly to regular curved surfaces.^23^ Still, knowledge of these dosimetric effects can be helpful in clinical cases in which curvature is present.

It is essential to understand the dosimetry under curved surfaces when using accurate absolute dose calculation algorithms because the monitor unit (MU) calculation from an accurate algorithm may disagree with the simple calculation performed by an MU verification system (MUV).^24^ This is because MUV calculations usually apply data collected in a phantom with a flat surface as part of commissioning the beam and treatment planning system (TPS).^3^ This aspect of simple MUV systems can be useful because the calculation is performed completely independently of issues with the patient data set. Still, disagreements between calculations need to be addressed by the clinical user if they exceed a set threshold.^24^ Causes of disagreement can include curvature, contour density overrides from the planning data set, inhomogeneity, sharp features of the electron cutout, calculation parameter choices, and more. Also, in the presence of a curved patient surface, the PDD (percent of dose vs. depth, normalized to the maximum dose) along the central axis can differ significantly from that in a flat‐surface water phantom.

The first purpose of this paper is to compare how the PDDs in curved and flat surfaces differ for treatment planning purposes. As a result, the TPS needs to calculate the dose using an accurate dose calculation algorithm. Accurate algorithms, such as Monte Carlo (MC) and pencil‐beam redefinition algorithms, have been evaluated by Hogstrom et al.^25^ The second purpose of this paper is to compare the ratio of dose output (cGy/MU) on the central axis at the depth of maximum dose (dmax) between calculations involving curved surface geometries and flat surfaces. This ratio can be used as a correction factor, which makes a simple MUV calculation more accurate.

## 2 MATERIALS AND METHODS

### 2.1 Treatment unit and TPS

The modeled treatment unit is a Varian IX medical linear accelerator (Varian Medical Systems, Palo Alto, CA) with electron energies of 6, 9, 12, 16, and 20 MeV, and applicator sizes A06, A10, A15, A20, and A25, where the number following the letter “A” represents the length, in cm, of the side of the square field at the isocenter.

The Varian Eclipse electron Monte Carlo (eMC) v15.606 algorithm was used for calculations. The algorithm represents a clinically commissioned algorithm so the results can be interpreted as clinically meaningful. Previous studies have validated the accuracy of this algorithm.^26^, ^27^, ^28^ Reference conditions were set so 100 MU equates to 100 cGy in water at a source‐to‐surface distance (SSD) of 100 cm for the A10 (10 cm × 10 cm) applicator at the respective dmax for each energy. Beam models were calculated using 1% statistical uncertainty.

### 2.2 Phantom data and field calculations

**FIGURE 2:**
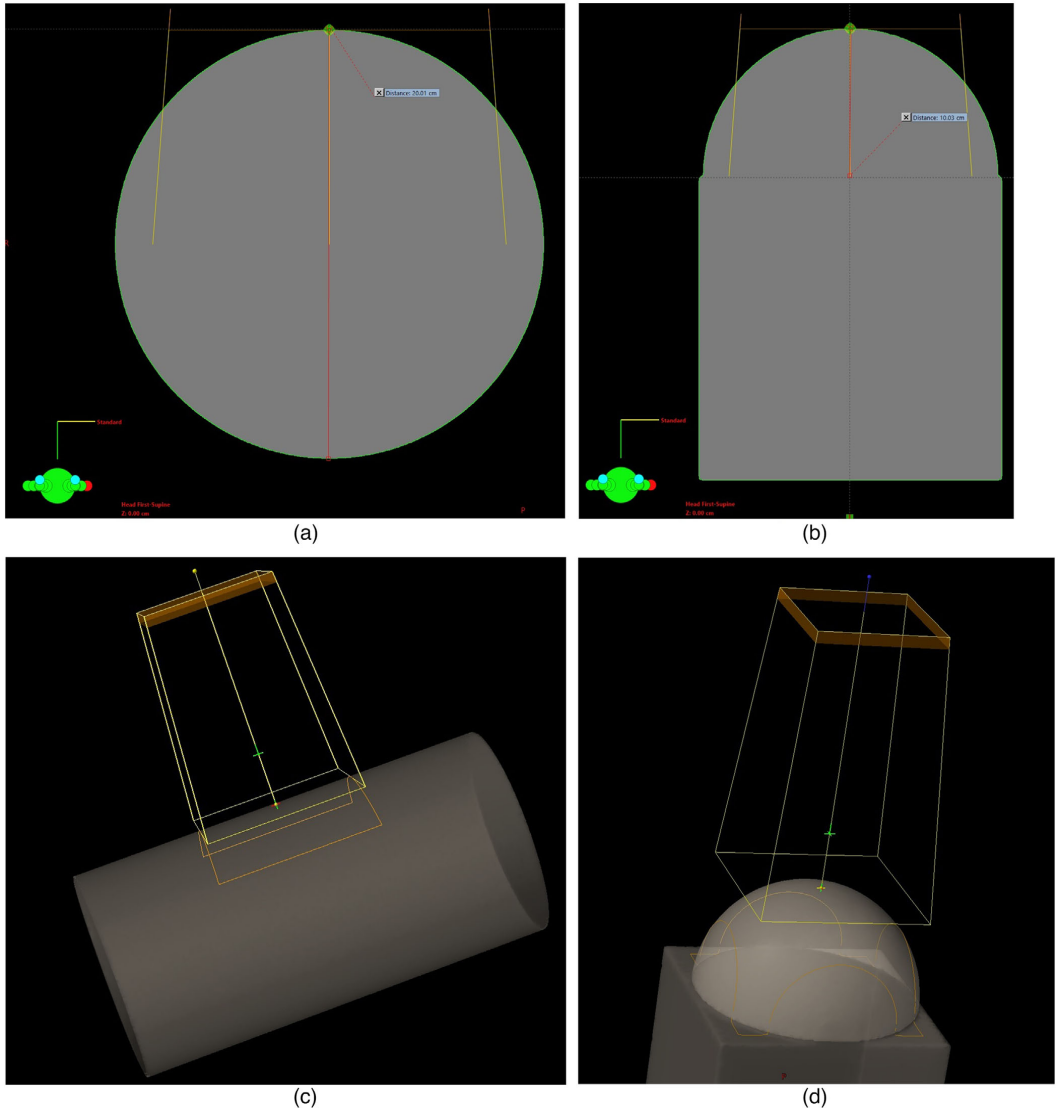
(a–d): (a) and (b) show axial slices of cylindrical *r* = 10 cm and spherical *r* = 10 cm phantoms, respectively. Measurement lines are drawn to show the radius of curvature of each phantom. (c) and (d) show the 3‐dimensional renderings of the beams incident to the surfaces of the phantoms from above.

A 40 cm × 40 cm × 40 cm cube, or “flat,” phantom was created in the TPS for calculating fields under measurement conditions. Six cylindrical phantoms with radii of curvature (*r*) of 2.5, 4, 6, 10, 15, and 20 cm, and two phantoms with hemispherical surfaces (referred to as “spherical phantoms”) with *r* = 4 and 10 cm were created to represent common treatment sites (Table 1 and Figures 1 and 2). The length of the cylindrical phantoms was set to 40 cm. The mass densities of all phantoms were set to 1.00 g/cm^3^, equivalent to water.

Fields were placed on each phantom, using all available energies and applicators up to the first applicator larger than the diameter of the phantom (Figure 2). Applicators two sizes larger than the phantom were not calculated; the exception is the *r* = 2.5 cm cylinder, which was calculated with the A06 and A10 applicators for consistency (Section 2.3.2). An open cutout accessory was added to each applicator. The central axis of each field was set to be perpendicularly incident (enface) to the center of the phantom's surface at 100 cm SSD. The eMC calculation grids were set to encompass the entirety of each phantom. Each field was assigned 100 MU.

The eMC calculation parameters were set to 1% statistical uncertainty, 0.2 cm calculation grid (voxel size), 3% statistical limit, and no smoothing. The parameters were selected to balance calculation time and precision.^26^

### 2.3 Methods of analysis

#### 2.3.1 Relative output factors (ROFs)

The point doses (outputs) calculated by eMC for 100 MU at dmax along the central axis were extracted from the TPS for each applicator, energy, and phantom combination. Outputs from voxels located 0.3 cm to the right and to the left of the central axis at dmax, where the dose can be assumed to be the same as the central‐axis dose, were also extracted. The doses from these three adjacent voxels were averaged together to mitigate the statistical uncertainty associated with the dose to one voxel. Relative output factors (ROFs) were created by dividing the average eMC calculated field output in the phantom (calculated output) by the output calculated using applicator factors measured at the time of commissioning (measured output). ROFs were calculated for fields incident on flat phantoms (ROF_Flat_) and curved phantoms (ROF_C_). ROF_Flat_ are expected to equal 1.000, which would indicate a valid beam model at dmax along the central axis.

##### Uncertainty in the ROFs

The uncertainty in the ROF data is determined by adding 1% from the beam model agreement with measured data to the statistical uncertainty of the field calculation, 1%, in each of the three dose voxels used to find the average ROF:

(1) $$\mathrm{ROF}\;\mathrm{uncertainty}\;=\;1\%\;+\frac{1\%}{\sqrt{3\;}}=\;1.6\%$$

The uncertainty value of ± 0.016 applies to ROF_Flat_ and ROF_C_.

#### 2.3.2 PDD data

Central‐axis PDD data from the A10 applicator were extracted from the TPS for each phantom and energy combination, normalizing the maximum dose along each curve to 100%. PDDs from other applicators were not evaluated. Some PDD curves extracted from beams at higher energies and in smaller phantoms are abridged because of the small phantom sizes.

The terms “R100,” “R90,” and “R50” are used to indicate the respective depths of the 100%, 90%, and 50% PDD points, similar to the terminology used in the report from AAPM TG‐25.^1^ The term “R100” refers to the same point as “dmax,” which is used to discuss the ROF.

## 3 RESULTS

### 3.1 Relative output factors (ROFs)

Table 2 shows the ROFs for each phantom, applicator, and beam energy combination. Decreases of 0%−10% are observed in cylindrical phantoms, with the largest differences in *r* = 2.5 and 4 cm. Differences of 2%−14% are observed in spherical phantoms with radii of *r* = 4 and 10 cm. ROF changes are dependent on a combination of beam energy, radius of curvature, and field size. Greater ROF decreases were observed in phantoms with more curvature, for the same energy and applicator combination. Overall, the largest ROF decreases were observed at low‐ and mid‐energy beams (6–12 MeV), whereas ROFs at higher energies (16 and 20 MeV) were less affected by curvature.

**TABLE 2:** Relative output factors (ROFs) for phantom, energy, and applicator combinations.

**Energy (MeV)**		**ROF of flat phantom**	**ROF of cylindrical phantoms—listed by radius of curvature (cm)**						**ROF of spherical phantoms by radius (cm)**	
	**Applicator**		**20**	**15**	**10**	**6**	**4**	**2.5**	**10**	**4**
**6**	**A6**	0.987	1.002	0.987	0.978	0.982	0.948	0.912	0.942	0.889
	**A10**	1.001	0.984	0.990	0.987	0.968	0.965	0.923	0.950	0.885
	**A15**	0.991	0.983	0.996	0.978	0.983	N/A	N/A	0.968	N/A
	**A20**	1.013	1.011	0.995	1.002	N/A	N/A	N/A	0.963	N/A
	**A25**	1.004	0.978	0.987	N/A	N/A	N/A	N/A	N/A	N/A
										
**9**	**A6**	0.992	0.995	0.995	0.975	0.953	0.942	0.903	0.952	0.879
	**A10**	0.992	1.004	0.975	0.975	0.946	0.939	0.902	0.963	0.864
	**A15**	1.001	0.989	0.985	0.984	0.953	N/A	N/A	0.951	N/A
	**A20**	0.997	0.997	0.995	0.986	N/A	N/A	N/A	0.970	N/A
	**A25**	0.989	0.993	0.988	N/A	N/A	N/A	N/A	N/A	N/A
										
**12**	**A6**	1.015	0.986	0.980	0.971	0.962	0.944	0.916	0.957	0.888
	**A10**	1.009	1.003	1.008	0.982	0.971	0.942	0.889	0.949	0.867
	**A15**	1.010	0.999	0.992	0.976	0.961	N/A	N/A	0.967	N/A
	**A20**	1.009	0.997	0.982	0.976	N/A	N/A	N/A	0.938	N/A
	**A25**	0.996	0.991	1.000	N/A	N/A	N/A	N/A	N/A	N/A
										
**16**	**A6**	0.989	0.978	0.981	0.977	0.979	0.965	0.934	0.954	0.919
	**A10**	0.994	0.985	0.997	0.977	0.965	0.957	0.925	0.966	0.902
	**A15**	1.003	0.991	0.996	0.982	0.975	N/A	N/A	0.948	N/A
	**A20**	1.001	1.014	0.989	0.990	N/A	N/A	N/A	0.960	N/A
	**A25**	1.005	0.994	0.988	N/A	N/A	N/A	N/A	N/A	N/A
										
**20**	**A6**	0.999	0.998	1.001	0.990	0.990	0.981	0.989	0.985	0.964
	**A10**	0.997	0.999	0.996	0.987	0.999	0.979	0.974	0.982	0.959
	**A15**	0.984	0.994	0.990	1.006	0.985	N/A	N/A	0.983	N/A
	**A20**	1.003	1.000	0.985	0.999	N/A	N/A	N/A	0.977	N/A
	**A25**	0.997	0.994	1.003	N/A	N/A	N/A	N/A	N/A	N/A

ROF = TPS/Measured. ROF uncertainty: ± 0.016.

### 3.2 Analysis of PDDs

**FIGURE 3:**
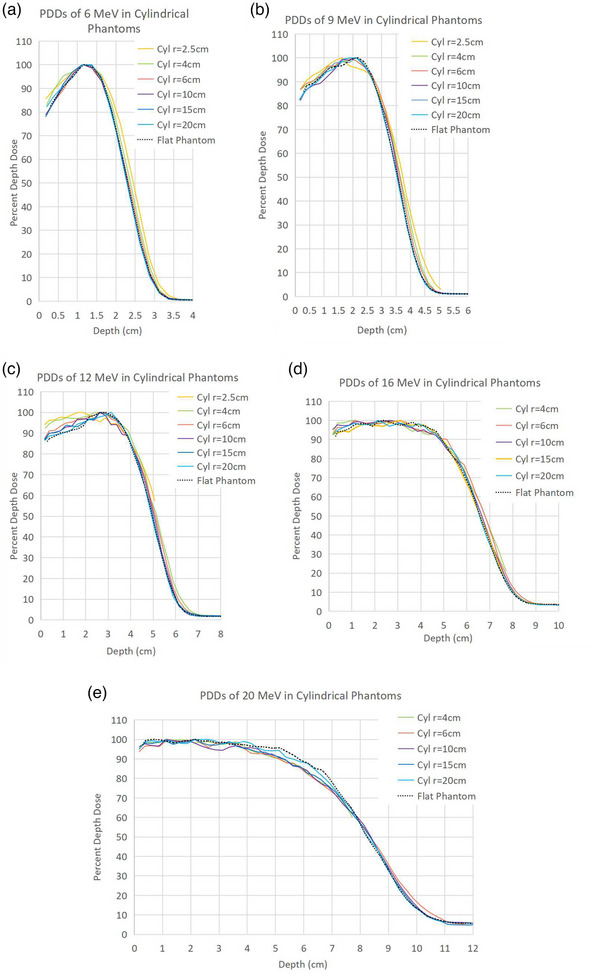
(a–e): PDDs in cylindrical phantoms. Each curve is normalized such that the maximum dose equals 100%.

**FIGURE 4:**
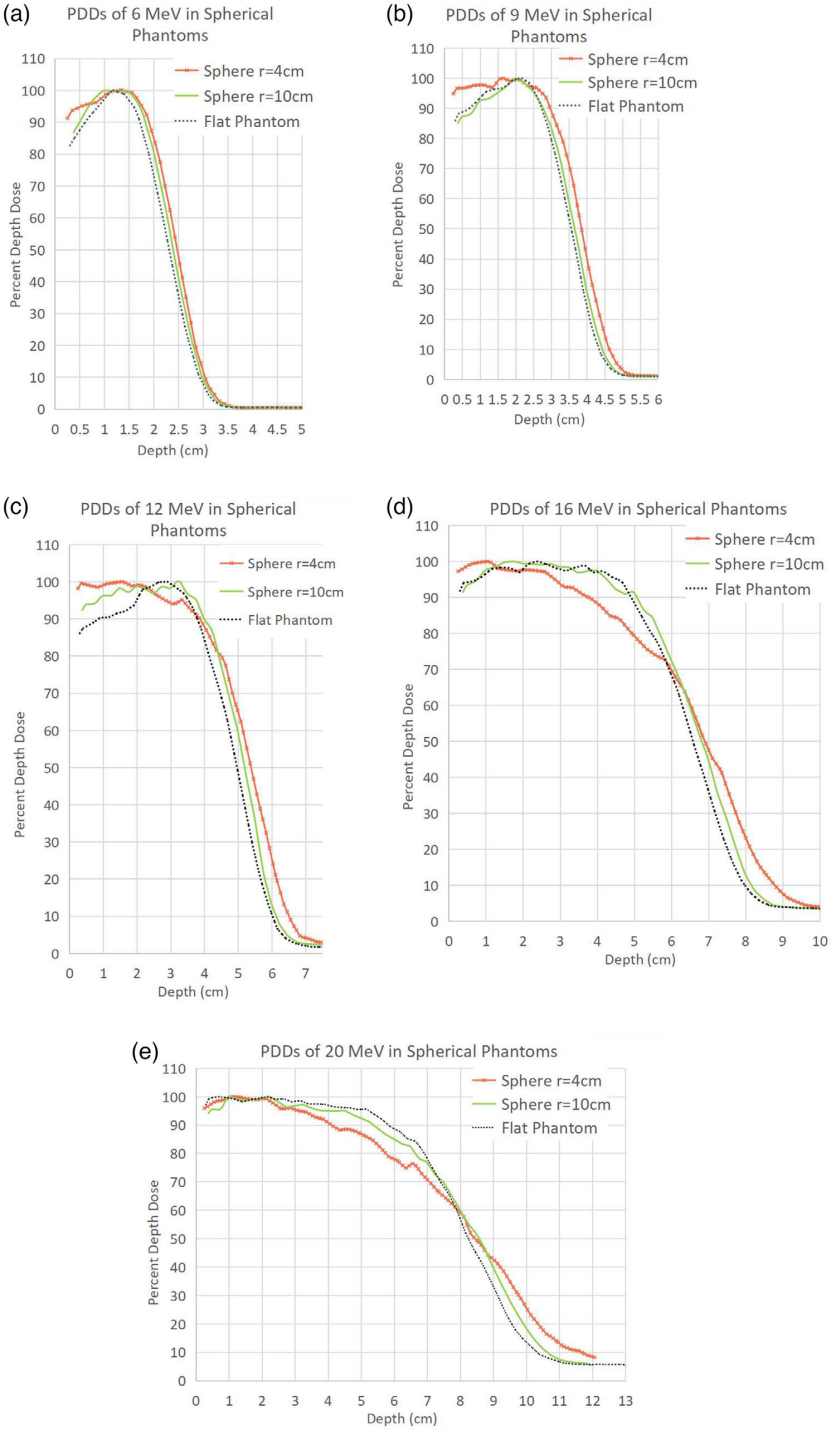
(a–e): PDDs in spherical phantoms. Each curve is normalized such that the maximum dose equals 100%.

Figures 3(a–e) and 4(a–e) show PDD data from the A10 applicator at each energy in cylindrical and spherical phantoms, respectively.

#### 3.2.1 Cylindrical phantom PDDs

The entrance dose was observed to increase between the surface and R100 at all energies, up to 5% for *r* = 4 cm and 10% for *r* = 2.5 cm. This effect was enhanced closer to the surface and at lower energies. For 6, 9, and 12 MeV, the central‐axis PDDs were almost identical beyond R100 compared to the PDDs in flat phantoms.

In addition to the observations at lower energies, R100 and distal R90 shift upstream in cylinders *r* = 10, *r* = 6, and *r* = 4 cm for energies of 16 and 20 MeV. PDD data were not compiled for these energies in the *r* = 2.5 cm phantom. An upstream shift of distal R90 was also observed at 12 MeV in *r* = 2.5 cm. The shifts were less than 1.0 cm, compared to R90 in the flat phantom. R100 was observed to shift upstream by less than 0.2 cm at energies for which R90 was relatively unchanged. This effect was slightly enhanced with decreasing phantom radius. R50 values remained almost identical for all energies and phantoms; the greatest changes to R50 were observed to be shifts downstream of less than 0.3 cm in the smaller phantoms at the mid energies.

#### 3.2.2 Spherical phantom PDDs

Peak broadening was observed between the proximal R90 and distal R90, and the entrance dose increased 5%−12% compared to that of the flat phantom. Both effects were enhanced at lower energies. As with cylindrical phantoms, R90 was observed to shift upstream more than 0.5 cm at higher energies and in the smaller phantom. Lastly, R50 shifted downstream by 0.5 cm or less; a larger shift was observed in smaller phantoms. This last effect was not observed to the same degree in cylindrical phantoms and likely is more observable in spherical phantoms because of curvature in two dimensions.

## 4 DISCUSSION

### 4.1 Comparison of results with prior data

Ritenour et al. published ionization ratios from measurements taken at different depths beneath various cylindrical water phantom surfaces. The measurements were performed in electron beams from a Clinac‐18 linear accelerator.^23^ Tables 3 and 4 display the portions of those data that help evaluate the results from this study. The ionization ratios comparing measurements of central‐axis points at the depth of dmax in cylindrical phantoms to points in flat‐surface phantoms agree with the ROF data calculated in this study within 1.7% (Table 3). Additionally, the ratios of the measured depth‐ionization values for the 18 MeV beam show the same trend of the distal falloff with curvature as seen in the 16 and 20 MeV PDD calculations: First, the ratio of the curved surface to flat surface decreases, then it increases at a deeper depth (Table 4).

**TABLE 3:** Comparison of calculated ROFs to prior data.

**Energy (MeV)**	**ROF (from** **Table** **2**)	**Ionization ratio, measured at dmax^a^**	**% Difference**
6	0.978	0.97	0.82
9	0.984	0.97	1.44
12	0.976	0.97	0.62
15/16	0.982 (16 MeV)	0.97 (15 MeV)	1.24
18/20	1.006 (20 MeV)	0.99 (18 MeV)	1.62

*r* = 10 cm cylinder, A15.

^a^ Reproduced with permission from Ritenour et al, Med Phys 1983, (Figure 3, off‐axis distance = 0 cm; data chosen from the approximate depths of energy‐specific dmax or expected flat portion of curves for higher energies. Ionization ratios are for curved surfaces relative to flat surfaces).^23^

**TABLE 4:** Prior distal falloff data, measured at 18 MeV in cylindrical phantoms.

Radius of curvature (cm)	Ionization ratio measured at distal portion of curve^a^	
	*d* = 4 cm	*d* = 7 cm
15	0.97	1.02
10	0.97	1.01
7.5	0.97	1.11
6	0.97	1.09

Increasing ionization ratios with depth show changes in distal falloff shape between flat‐ and curved‐surface measurements. A decreased ratio, followed by an increased ratio, is similar to the trend observed in 16 and 20 MeV PDD data calculated in this study. The effect is more observable in smaller phantoms.

^a^ Reproduced with permission from Ritenour et al, Med Phys 1983 (Figure 2, off‐axis distance = 0 cm; ionization ratios are for curved surfaces relative to flat surfaces in 15 × 15 cm^2^ field).^23^

### 4.2 Physical basis for PDD and ROF results

The central‐axis PDD in a curved‐surface phantom differs from that in a flat‐surface phantom due to air replacing phantom material lateral to the central axis. This effect, referred to as the “missing volume” or “missing material” effect, disrupts side‐scatter equilibrium, resulting in several effects on the central‐axis depth dose.^23^ First, without normalizing the depth doses to the dose at dmax, both phantoms have the same dose at the surface before side‐scatter differences take effect. As depth increases, some electrons that scatter to the central axis in the flat phantom cannot do so in the curved phantom due to air replacing phantom material. This decreases the central‐axis dose as depth increases, which results in lower doses at R100, R90, the shoulder, and the first portion of the distal falloff region after R90. Second, at depths near R50 and beyond, the dose in the curved‐surface phantom becomes greater than that in the flat‐surface phantom because the electrons scattered to the central axis reach the end of their range at shallower depths in the flat‐surface phantom. Third, because the dose is reduced in the shallower portion of the PDD's distal falloff region but then is increased at greater depths, the two PDD curves will intersect in the distal falloff region near R50. Lastly, when the PDD curve is re‐normalized, so the new maximum dose equals 100%, the PDD between the surface and dmax is observed to increase, compared to PDD of the flat‐surface phantom at the same depths. These four effects vary in magnitude depending on beam energy, depth, and surface curvature.

Scattering power, which has an inverse relationship with the electron beam energy, explains the trend of larger ROF changes as well as larger changes in the PDD between the surface and dmax at lower energies when compared to higher energies in the same phantoms.^29^ Overall, the effects observed in cylindrical phantoms are more enhanced in spherical phantoms due to increased curvature.

### 4.3 Utilizing ROF values for second check of eMC dose calculation

This study shows how eMC accounts for curvature in the calculation of the PDDs and central‐axis doses at dmax. The dose calculations performed by eMC in this paper are “absolute dose calculations” that compute and plot absolute doses based on the MU setting in the TPS; the MU setting can be scaled by adjusting the prescription or normalization method. The inputs into eMC include the clinical CT dataset, structure set and any density overrides, and the clinical field parameters. To perform an independent second check, most MUV systems use tabulated commissioning data acquired from flat‐phantom measurements, with corrections available for field size, shape, and SSD. As a result of surface curvature, clinical users may experience a discrepancy between the MU calculated with accurate TPS dose calculations and simple MUV calculations for the same prescription dose.

In such “second check” scenarios, it is recommended that the clinical user corrects the MUV calculation using a curvature correction factor. A correction factor can be applied by using ROF values directly from Table 2 or by interpolating between values. For example, to correct the MUV system's calculation of the MU needed to achieve a prescribed dose to dmax, the user would divide the MUV result by the ROF. Additionally, a medical physicist can generate an entire reference set of ROFs for various curved surfaces, field sizes, and energy combinations using their institution's algorithm and accelerator data. If necessary, clinical users can address unresolved discrepancies for unique situations by determining a curvature correction factor using the method described in this paper: The user would create a curved‐surface phantom with a radius close to the clinical radius and calculate the clinical field parameters on the curved‐surface phantom and a flat‐surface phantom to determine the ROF.

## 5 CONCLUSION

Two‐ and three‐dimensional surface curvature affects the electron beam dose distribution calculated with the accurate dose algorithm eMC v15.6, compared to the distribution for a beam incident upon a flat surface. Dosimetric changes are dependent on the beam energy, applicator size, and radius of curvature of the phantom's surface. The dose outputs at dmax decreased 0%−14%, and several features of the PDDs changed. Treatment sites with a radius of curvature of 10 cm or less, such as the scalp, nose, neck, and extremities are affected the most by these dosimetric changes. To mitigate clinical discrepancies between simple MUV calculations and accurate TPS MU calculations, it is recommended to apply a correction factor to account for the effects of curvature. Curvature correction factors can be applied to any MUV calculation that uses tabulated data from a flat‐surface phantom.

## AUTHOR CONTRIBUTIONS

The sole author is responsible for all aspects of the paper including the inception, design, and completion of the experiment, the analysis and presentation of the data, as well as the drafting, editing, revising, and approval of the manuscript.

## CONFLICT OF INTEREST STATEMENT

The author declares no conflicts of interest.

## References

[1] Khan FM , Doppke KP , Hogstrom KR , et al. Clinical electron‐beam dosimetry: report of AAPM radiation therapy committee task group no. 25. Med Phys. 1991;18:73‐109. 10.1118/1.596695 1901132

[2] Gerbi BJ , Antolak JA , Deibel FC , et al. Recommendations for clinical electron beam dosimetry: supplement to the recommendations of task group 25. Med Phys. 2009;36:3239‐3279. 10.1118/1.3125820 19673223

[3] Das IJ , Cheng C‐W , Watts RJ , et al. Accelerator beam data commissioning equipment and procedures: report of the TG‐106 therapy physics committee of the AAPM. Med Phys. 2008;35:4186‐4215. 10.1118/1.2969070 18841871

[4] Almond PR , Biggs PJ , Coursey BM , et al. AAPM's TG‐51 protocol for clinical reference dosimetry of high‐energy photon and electron beams. Med Phys. 1999;26:1847‐1870. 10.1118/1.598691 10505874

[5] International Atomic Energy Agency (IAEA) . Absorbed Dose Determination In External Beam Radiotherapy: An International Code of Practice for Dosimetry Based on Standards of Absorbed Dose to Water. Vienna, Austria. Technical Report Series No. 398 (TRS‐398). 2000.

[6] Geurts MW , Jacqmin DJ , Jones LE , et al. AAPM MEDICAL PHYSICS PRACTICE GUIDELINE 5.b:commissioning and QA of treatment planning dose calculations—Megavoltage photon and electron beams. J Appl Clin Med Phys. 2022;23:e13641. 10.1002/acm2.13641 35950259 PMC9512346

[7] Perkins GH , McNeese MD , Antolak JA , Buchholz TA , Strom EA , Hogstrom KR . A custom three‐dimensional electron bolus technique for optimization of postmastectomy irradiation. Int J Radiat Oncol Biol Phys. 2001;51(4):1142‐1151. 10.1016/s0360-3016(01)01744-8 11704339

[8] DotDecimal DabajaB , Kudchadker R , Bolus Electron Conformal Therapy: Scalp. Accessed Jan 28, 2023.https://dotdecimal.com/wp‐content/uploads/2015/10/MD‐Anderson‐VTB‐Scalp.pdf

[9] Hogstrom KR , Mills MD , Eyer JA , et al. Dosimetric evaluation of a pencil‐beam algorithm for electrons employing a two‐dimensional heterogeneity correction. Int J Radiat Oncol Biol Phys. 1984;10(4):561‐596. 6725043 10.1016/0360-3016(84)90036-1

[10] Carver RL , Hogstrom KR , Chu C , Fields RS , Sprunger CP . Accuracy of pencil‐beam redefinition algorithm dose calculations in patient‐like cylindrical phantoms for bolus electron conformal therapy. Med Phys. 2013;40(7):071720. 10.1118/1.4811104 23822424

[11] Chen YL , DeLaney TF . Soft tissue and bone sarcomas. In: Khan FM , Gibbons JP , Sperduto PW , eds. Khan's Treatment Planning in Radiation Oncology. 4th ed. Wolters Kluwer; 2016:1057‐1100.

[12] Wooden KK , Hogstrom KR , Blum P , Gastorf RJ , Cox JD . Whole‐Limb irradiation of the lower calf using a six‐field electron technique. Med Dos. 1996;21(4):211‐218. 10.1016/S0958-3947(96)00129-X 8985926

[13] Cahlon O , Riaz N , Lee NY . Larynx cancer. In: Lee NY , Lu JJ , eds. Target Volume Delineation and Field Setup: A Practical Guide for Conformal and Intensity‐Modulated Radiation Therapy. Heidelberg Springer; 2013:21‐28. 10.1007/978-3-642-28860-9

[14] Wang X , Zhang N , Yu C , et al. Evaluation of neck circumference as a predictor of central obesity and insulin resistance in Chinese adults. Int J Clin Exp Med. 2015;8(10):19107‐19113. 26770540 PMC4694440

[15] Katz SL , Vaccani JP , Clarke J , Hoey L , Colley RC , Barrowman NJ . Creation of a reference dataset of neck sizes in children: standardizing a potential new tool for prediction of obesity‐associated diseases? BMC Pediatr. 2014;14:159. 10.1186/1471-2431-14-159 24952386 PMC4110068

[16] Kudchadker RJ , Antolak JA , Morrison WH , Wong PF , Hogstrom KR . Utilization of custom electron bolus in head and neck radiotherapy. J Appl Clin Med Phys. 2003;4(4):321‐333. 14604422 10.1120/jacmp.v4i4.2503 PMC5724465

[17] Ekstrand KE , Dixon RL . The problem of obliquely incident beams in electron‐beam treatment planning. Med Phys. 1982;9(2):276‐278. 10.1118/1.595084 6806597

[18] Antolak JA . Electron beam treatment planning. In: Khan FM , Sperduto PW , Gibbons JP , eds. Khan's Treatment Planning in Radiation Oncology. 5th ed. Lippincott Williams & Wilkins, Wolters Kluwer Health; 2021:665‐689.

[19] Hogstrom KR , Evaluation of electron pencil beam dose calculations. In: Kereiakes JG , Elson HR , Born CG , eds. Radiation Oncology Physics ‐ 1986: Proceedings of the 1986 Summer School of the AAPM. American Institute of Physics;1987:532‐557.

[20] Morrison WH , Wong PF , Starkschall G , et al. Water bolus for electron irradiation of the ear canal. Int J Radiat Oncol Biol Phys. 1995;33(2):479‐483. 10.1016/0360-3016(95)00023-r 7673037

[21] Kudchadker RJ , Hogstrom KR , Garden AS , McNeese MD , Boyd RA , Antolak JA . Electron conformal radiotherapy using bolus and intensity modulation. Int J Radiat Oncol Biol Phys. 2002;53(4):1023‐1037. 10.1016/s0360-3016(02)02811-0 12095572

[22] Ulin K , Palisca M . The use of scattering foil compensators in electron beam therapy. Int J Radiat Oncol Biol Phys. 1996;35(4):785‐792. 10.1016/0360-3016(96)00153-8 8690646

[23] Ritenour ER , Cacak RK , Hendee WR . Ionization produced by electron beams beneath curved surfaces. Med Phys. 1983;10:669‐671. 10.1118/1.595334 6646073

[24] Stern RL , Heaton R , Fraser MW , et al. Verification of monitor unit calculations for non‐IMRT clinical radiotherapy: report of AAPM task group 114. Med Phys. 2011;38:504‐530. 10.1118/1.3521473 21361219

[25] Hogstrom KR , Pitcher GM , Carver RL , Antolak JA . Treatment planning algorithms: electron beams. In: Khan FM , Sperduto PW , Gibbons JP , eds. Khan's Treatment Planning in Radiation Oncology. 5th ed. Lippincott Williams & Wilkins, Wolters Kluwer Health; 2021:467‐495.

[26] Popple RA , Weinberg R , Antolak JA , et al. Comprehensive evaluation of a commercial macro Monte Carlo electron dose calculation implementation using a standard verification data set. Med Phys. 2006;33(6Part1):1540‐1551. 10.1118/1.2198328 16872061

[27] Zhang A , Wen N , Nurushev T , Burmeister J , Chetty IJ . Comprehensive evaluation and clinical implementation of commercially available Monte Carlo dose calculation algorithm. J Appl Clin Med Phys. 2013;14:127‐145. 10.1120/jacmp.v14i2.4062 23470937 PMC5714370

[28] Carver RL , Sprunger CP , Hogstrom KR , Popple RA , Antolak JA . Evaluation of the Eclipse eMC algorithm for bolus electron conformal therapy using a standard verification dataset. J Appl Clin Med Phys. 2016;17(3):52‐60. 10.1120/jacmp.v17i3.5885 27167259 PMC5690899

[29] Khan FM , Gibbons JP . Khan's the Physics of Radiation Therapy. 4th ed. Lippincott Williams and Wilkin; 2014:258.

